# GPER activation protects against epithelial barrier disruption by ***Staphylococcus****aureus* α-toxin

**DOI:** 10.1038/s41598-018-37951-3

**Published:** 2019-02-04

**Authors:** Kathleen D. Triplett, Srijana Pokhrel, Moriah J. Castleman, Seth M. Daly, Bradley O. Elmore, Jason A. Joyner, Geetanjali Sharma, Guy Herbert, Matthew J. Campen, Helen J. Hathaway, Eric R. Prossnitz, Pamela R. Hall

**Affiliations:** 1 University of New Mexico College of Pharmacy, Department of Pharmaceutical Sciences, Albuquerque, NM 87131 USA; 2 University of New Mexico School of Medicine, Department of Internal Medicine, Albuquerque, NM 87131 USA; 3 University of New Mexico School of Medicine, Department of Cell Biology & Physiology, Albuquerque, NM 87131 USA

## Abstract

Sex bias in innate defense against *Staphylococcus aureus* skin and soft tissue infection (SSTI) is dependent on both estrogen production by the host and *S. aureus* secretion of the virulence factor, α-hemolysin (Hla). The impact of estrogen signaling on the immune system is most often studied in terms of the nuclear estrogen receptors ERα and ERβ. However, the potential contribution of the G protein-coupled estrogen receptor (GPER) to innate defense against infectious disease, particularly with respect to skin infection, has not been addressed. Using a murine model of SSTI, we found that GPER activation with the highly selective agonist G-1 limits *S. aureus* SSTI and Hla-mediated pathogenesis, effects that were absent in GPER knockout mice. Specifically, G-1 reduced Hla-mediated skin lesion formation and pro-inflammatory cytokine production, while increasing bacterial clearance. *In vitro*, G-1 reduced surface expression of the Hla receptor, ADAM10, in a human keratinocyte cell line and increased resistance to Hla-mediated permeability barrier disruption. This novel role for GPER activation in skin innate defense against infectious disease suggests that G-1 may have clinical utility in patients with epithelial permeability barrier dysfunction or who are otherwise at increased risk of *S. aureus* infection, including those with atopic dermatitis or cancer.

## Introduction

*Staphylococcus aureus* is the primary cause of skin and soft tissue infection (SSTI) worldwide^1–3^. In the U.S., more than half of the isolates are methicillin-resistant (MRSA) strains, limiting antibiotic treatment strategies^1,2^. The skin permeability barrier serves as the first line of defense against external insults such as bacterial pathogens^4,5^, still the cost of treating SSTI reaches billions of dollars annually^6^. To breach epithelial barriers, the majority of *S. aureus* isolates secrete the pore-forming toxin, alpha-hemolysin (Hla)^7^. Hla facilitates invasive infection by hijacking the host molecule ADAM10 (a disintegrin and metalloprotease 10) to disrupt cell junctions and thus host permeability barriers^7–16^. Since Hla-mediated epithelial injury controls infection outcome^17^, numerous prophylactic and therapeutic strategies to directly target Hla are being pursued as treatment options^8,18–25^. Interestingly, we recently reported a sex bias in SA SSTI in male versus female patients^26^, and showed in a murine SSTI model that sex bias in *S. aureus* SSTI is driven by a sex- and estrogen-specific response to Hla^9,26^. This suggests that host-directed therapies (HDT) might be developed to limit invasive disease by protecting barrier integrity in the face of Hla-challenge.

Historically, estrogen has been known to exert its numerous effects on the immune response by signaling through the classical nuclear estrogen receptors ERα and ERβ^27^. More recently, the G protein-coupled estrogen receptor (GPER) has been recognized as mediating many of the rapid and even long-term effects of estrogen^28,29^. GPER activation has been shown to modulate macrophage cytokine production and neutrophil function^30–32^, as well as to reverse stroke-induced peripheral immunosuppression in ovariectomized mice^33^. Interestingly, GPER activation by the highly selective GPER agonist G-1^34^ has also been reported to block disruption of endothelial barrier integrity as shown by its ability to limit blood-brain barrier (BBB) disruption following global cerebral ischemia (GCI)^35^. In addition to endothelial cells, GPER is also expressed in numerous types of skin cells including keratinocytes, melanocytes and dermal fibroblasts^36–39^. However, the potential contribution of GPER activation to skin immunity, particularly with respect to innate defense against bacterial infection, has not been addressed. Therefore, given the role of *S. aureus* Hla in SSTI and disruption of epithelial cell junctions, we hypothesized that G-1-mediated activation of GPER would limit Hla-mediated epithelial permeability barrier disruption and reduce *S. aureus* pathogenesis.

To test this hypothesis, we used a murine model of SSTI^9^ to test whether G-1 limits *S. aureus* SSTI and Hla-mediated pathogenesis in a GPER-dependent manner. Specifically, G-1 treatment reduces Hla-mediated skin lesion formation and production of pro-inflammatory cytokines *in vivo*. Consistent with its ability to support BBB integrity following GCI^35^, G-1 treatment of a human keratinocyte cell line increased intercellular junction integrity in the face of Hla-mediated permeability barrier disruption. Furthermore, G-1 reduced keratinocyte surface expression of the Hla receptor, ADAM10, as well as E-cadherin cleavage with Hla-challenge, a mechanism that may contribute to the overall increase in permeability barrier integrity. Together, these studies clearly demonstrate a novel role for GPER activation in skin innate defense against *S. aureus* infection and the important virulence factor, Hla, as well as the potential of G-1 as an HDT to limit infectious disease.

## Results

### GPER activation reduces pathogenesis in a mouse model of *S. aureus* SSTI

**Figure 1:**
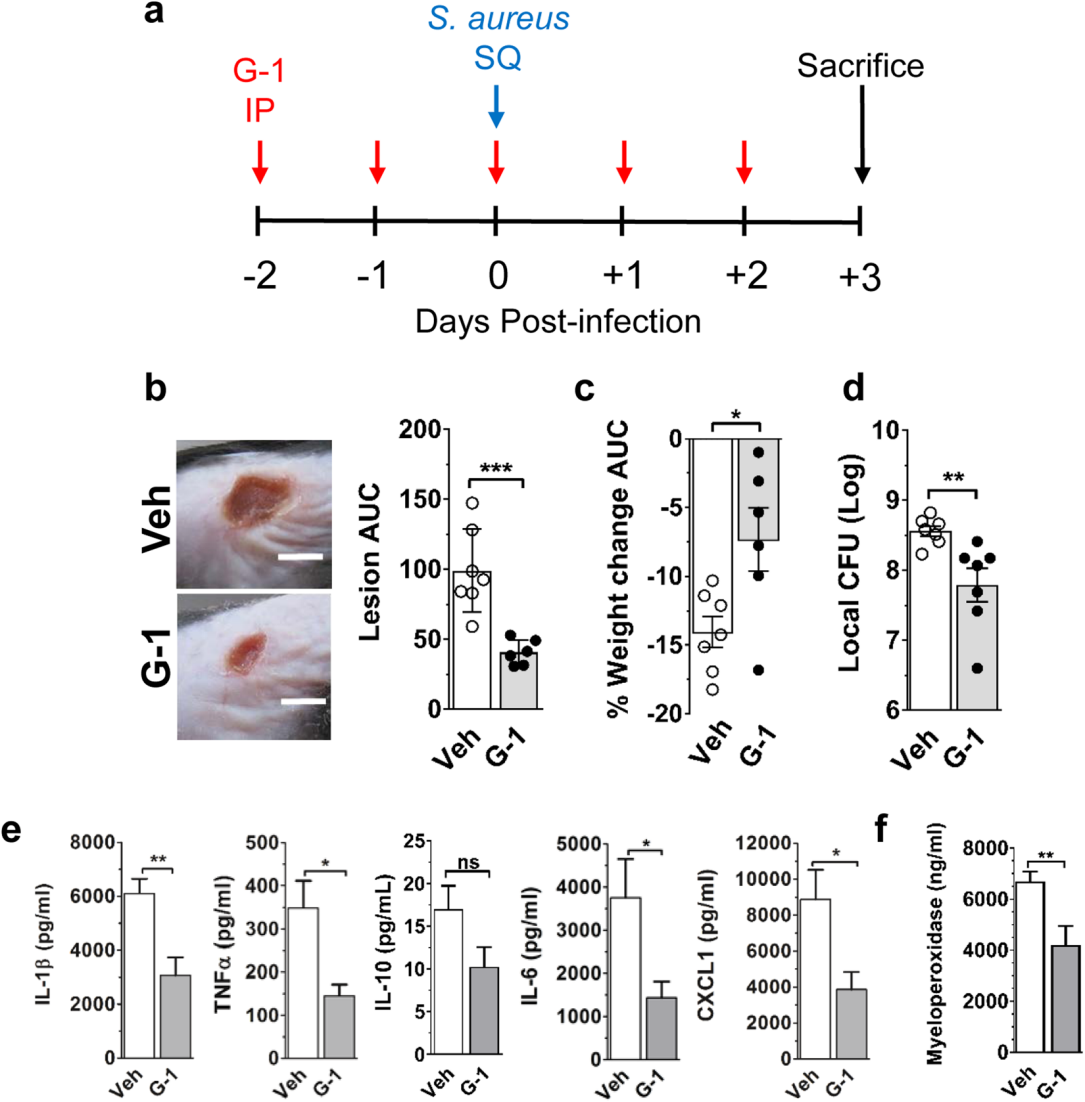
G-1 promotes protection against *S. aureus* SSTI in male mice. WT (C57BL/6J) male mice were treated IP with vehicle (Veh) or 200 ng G-1 on days −2, −1, 0, + 1 and + 2 relative to SQ infection with 2 × 10^7^ CFU of USA300 MRSA isolate LAC. Mice were weighed and lesion size measured daily before mice were sacrificed on day 3 post-infection. (**a**) Illustration of the G-1 treatment and infection timeline. (**b**) Representative day 3 post-infection images and area under the curve (AUC) for lesion size (mm^2^) and (**c**) percent weight change (relative to pre-infection weight) over the 3-day infection. (**d**) Day 3 post-infection bacterial burden. (**e**) IL-1β, TNFα, IL-6, CXCL1 and IL-10 levels and (**f**) myeloperoxidase levels in clarified injection site homogenate collected on day 3 from the site of infection. Data are mean ± SEM, n = 6–7 mice per group from two independent experiments. Unpaired t-test: ns, not significant; *p < 0.05; **p < 0.01; ***p < 0.001.

GPER activation has a variety of effects on innate immune function, including modulation of macrophage cytokine production and neutrophil function^30–32^, as well as reversing stroke-induced immunosuppression^33^. To determine whether GPER activation would support innate immune defense against infectious disease, we evaluated the effects of GPER activation on the outcomes of *S. aureus* infection using a well characterized murine model of SSTI^9^. Male mice were treated with the GPER-selective agonist G-1^34,40^ or vehicle control prior to subcutaneous (SQ) infection with the community-acquired MRSA isolate LAC^41^ (Fig. 1a). Over the course of a three-day infection, G-1-treated mice showed significantly reduced lesion area (neutrophil-filled abscesses with subsequent dermonecrosis) (p < 0.001) and weight loss (p < 0.05) (a general measure of morbidity) compared to vehicle-treated controls (Fig. 1b,c). On day 3 post-infection (typically the peak of lesion formation^42^), G-1-treated males also had reduced bacterial burden compared to control-treated mice (Fig. 1d). Consistent with reduced lesion area, bacterial burden, and the demonstrated anti-inflammatory effects of G-1^30^, G-1-treated mice also had lower local levels of the inflammatory cytokines IL-1β, TNFα, IL-6 and CXCL1 (Fig. 1e). As expected, given lower levels of the neutrophil-recruiting chemokine CXCL1, local levels of myeloperoxidase (MPO), often used as a surrogate marker for neutrophil presence^43^, were reduced in G-1-treated mice (Fig. 1f) suggesting a potential association between reduced lesion size with G-1-treatment and reduced neutrophil accumulation. In contrast to reduced levels of pro-inflammatory cytokines, levels of the anti-inflammatory cytokine IL-10 did not significantly differ between groups (p = 0.0884). This indicates that while G-1 reduces inflammation, it is not a general suppressor of cytokine production (Fig. 1e).

Female mice are innately better protected than males against *S. aureus* SSTI^26^, so we asked whether G-1 would further limit pathogenesis in female mice. At the male infectious dose of 2 × 10^7^ LAC CFU, females develop much smaller lesions than males. Therefore, to assess additional G-1 protection in females, the infectious dose was increased to ~3 × 10^7^ CFU. Compared to vehicle-treated controls, G-1-treated female mice showed significant reductions in lesion area (p < 0.01), whereas differences in weight loss and bacterial burden did not reach statistical significance (Supplementary Fig. S1). G-1-treated female mice also had reduced levels of the inflammatory cytokine TNFα at the site of infection, with no significant reduction in levels of the other cytokines tested or MPO compared to vehicle-treated mice (Supplementary Fig. S1). Given that G-1 does not directly inhibit bacterial growth (Supplementary Fig. S2), these results suggest that GPER activation limits the severity of *S. aureus* SSTI by a host-dependent mechanism.

### G-1 limits the severity of *S. aureus* SSTI in a GPER-dependent manner

**Figure 2:**
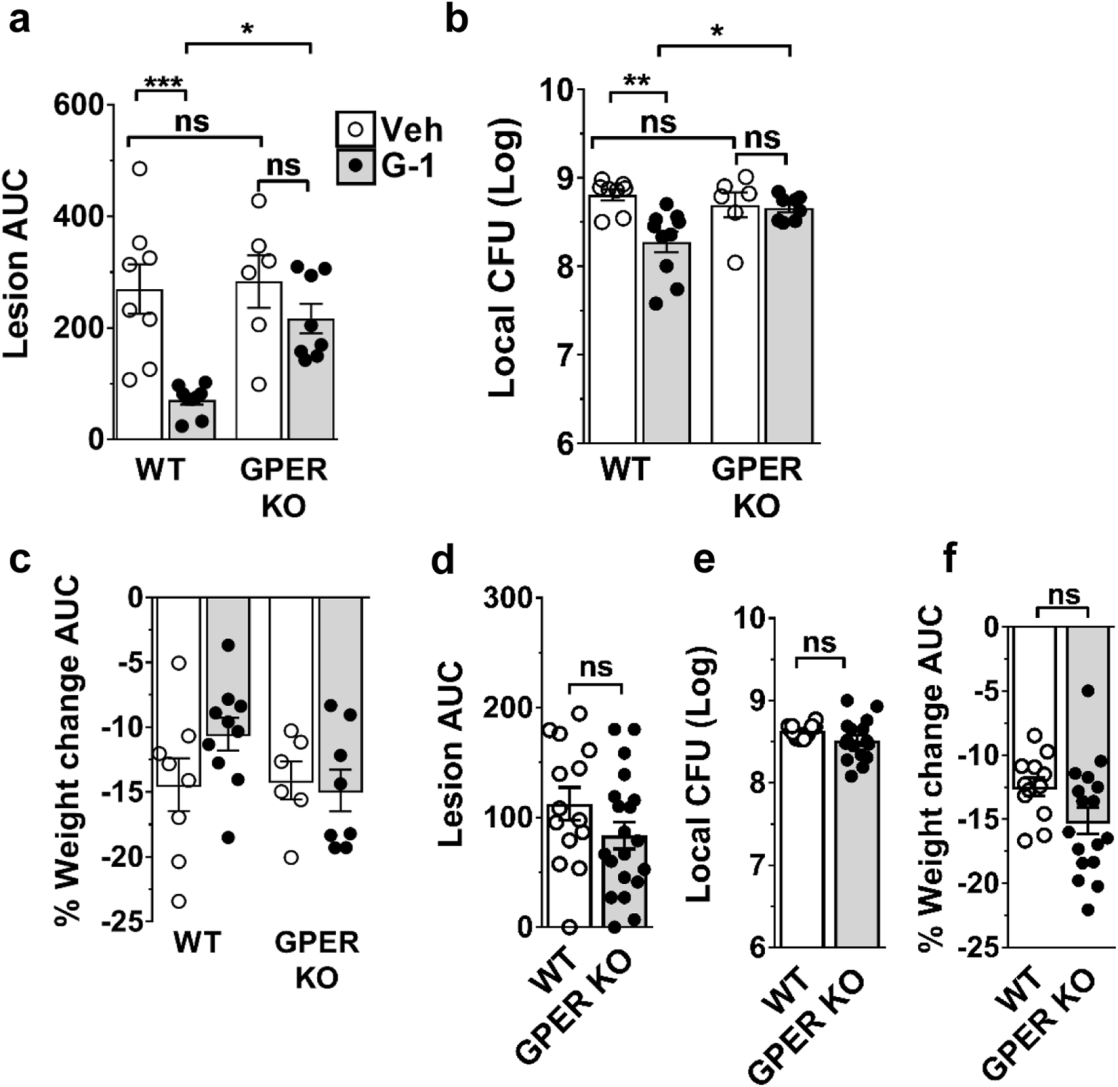
G-1-mediated protection against SSTI is GPER-dependent. (**a–c**) WT (C57BL/6) and GPER KO male mice were treated IP with vehicle or 200 ng G-1 on days −2, −1, 0, + 1 and + 2 relative to SQ infection with ~3 × 10^7^ CFU of USA300 MRSA isolate LAC. Shown are (**a**) lesion area (AUC in mm^2^) over the course of a 3-day infection, (**b**) day 3 post-infection bacterial burden at the site of infection and (**c**) percent weight change (AUC) post-infection. n = 6–9 mice per group from two independent experiments. (**d–f**) Female WT and GPER KO mice were infected as described above. Shown are (**d**) lesion AUC (mm^2^) over the course of a 3-day infection, (**e**) day 3 post-infection bacterial burden at the site of infection and (**f**) percent weight change (AUC) post-infection. n = 14–21 mice per group from three independent experiments. Data are mean ± SEM. ANOVA (**a**) p = 0.0002, (**b**) p = 0.0024 and (**c**) not significant. Tukey’s multiple comparison test (**a–c**) or Unpaired *t*-test (d-f): ns, not significant; *p < 0.05; **p < 0.01; ***p < 0.001.

G-1 is a highly selective GPER ligand with respect to both classical estrogen receptors and other GPCRs^30,34^, suggesting that the benefits of G-1-treatment against *S. aureus* SSTI should depend on host expression of GPER. To test this, we compared outcomes between male GPER knockout (GPER KO) mice and corresponding wild-type (WT) C57BL/6 controls treated with G-1 or vehicle and infected with LAC. Compared to vehicle-treated controls, G-1-treatment significantly reduced lesion size (p < 0.001) and increased bacterial clearance (p < 0.01) without affecting weight loss in infected WT mice, but had no effect on lesion size or bacterial clearance in GPER KO mice (Fig. 2a–c). Notably, infection outcomes in the absence of G-1 did not significantly differ between WT and GPER KO male (Fig. 2a–c) or female (Fig. 2d–f) mice. This indicates that innate defense in this model is normally GPER-independent and suggests that increased estrogen-dependent, innate protection in female mice^26^ is mediated through classical estrogen receptors. However, our findings clearly show that G-1 limits pathogenesis in both males and females and that GPER could be a promising target for HDT. Most importantly, these results demonstrate that the efficacy of G-1 in limiting *S. aureus* SSTI is host- and GPER-dependent.

### G-1 limits Hla-mediated pathogenesis in a murine dermonecrosis model

**Figure 3:**
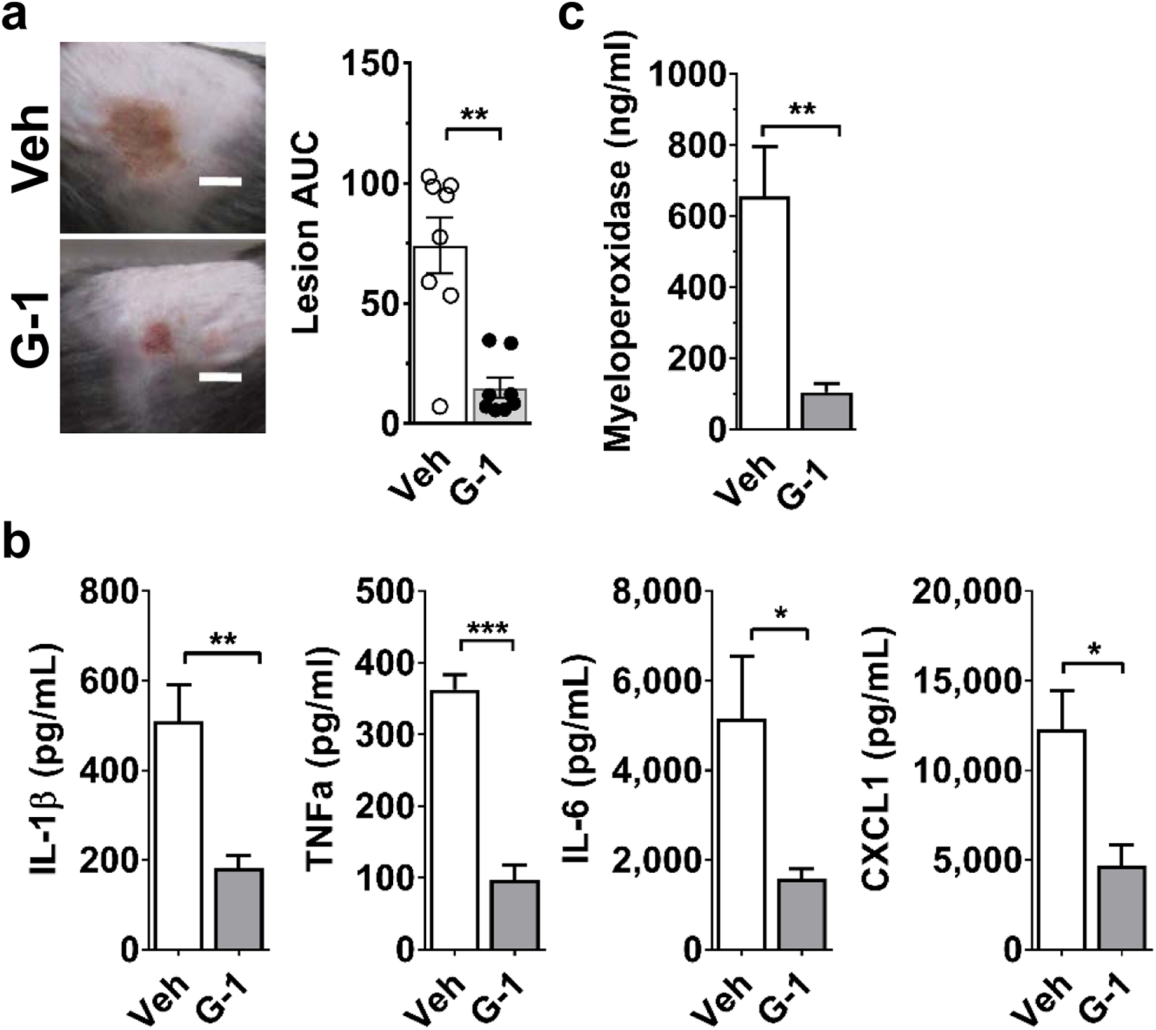
G-1 reduces Hla-mediated lesion formation and inflammation. WT (C57BL/6 J) male mice were treated IP with vehicle or 200 ng G-1 on days −2 through + 2 relative to SQ injection of 1 µg Hla. (**a**) Representative day 3 post-injection images (scale bar = 5 mm) and lesion size (AUC in mm^2^) over the 3 days post-injection. (**b**) IL-1β, TNFα, IL-6, CXCL1 and (**c**) myeloperoxidase levels measured in clarified injection site homogenate collected on day 3 post-injection. Data are mean ± SEM, n = 8 mice per group from two independent experiments. Mann –Whitney *U* test: *p < 0.05; **p < 0.01; ***p < 0.001.

In animal models of *S. aureus* SSTI, the secreted virulence factor alpha-hemolysin (Hla) drives lesion formation at the site of infection^8,10,11^. Given the host-dependent protective effects of G-1 against *S. aureus* SSTI, we postulated that G-1-treatment would limit pathogenesis in male mice directly challenged with Hla. Consistent with results in mice infected with *S. aureus*, G-1 treatment significantly reduced lesion formation, inflammatory cytokine production (IL-1β, TNFα, IL-6 and CXCL1) and MPO levels at the site of subcutaneous Hla-injection compared to vehicle-treated controls (Fig. 3). To corroborate the role of Hla in G-1-mediated protection in the skin, we infected male mice with an isogenic Hla deletion mutant of *S. aureus* (LACΔ*hla*). In the absence of Hla expression, skin lesion formation is minimal or absent, so outcomes are based largely on bacterial burden and weight loss. Importantly, G-1-treatment did not significantly alter LACΔ*hla* infection outcomes based on day three post-infection bacterial clearance and overall weight loss (Supplementary Fig. S3). Given that G-1 does not impact bacterial Hla expression or Hla activity (Supplementary Fig. S3), these results provide support for a mechanism in which G-1 protects against Hla-mediated pathogenesis by altering the host response.

### G-1 limits keratinocyte permeability barrier disruption by Hla

**Figure 4:**
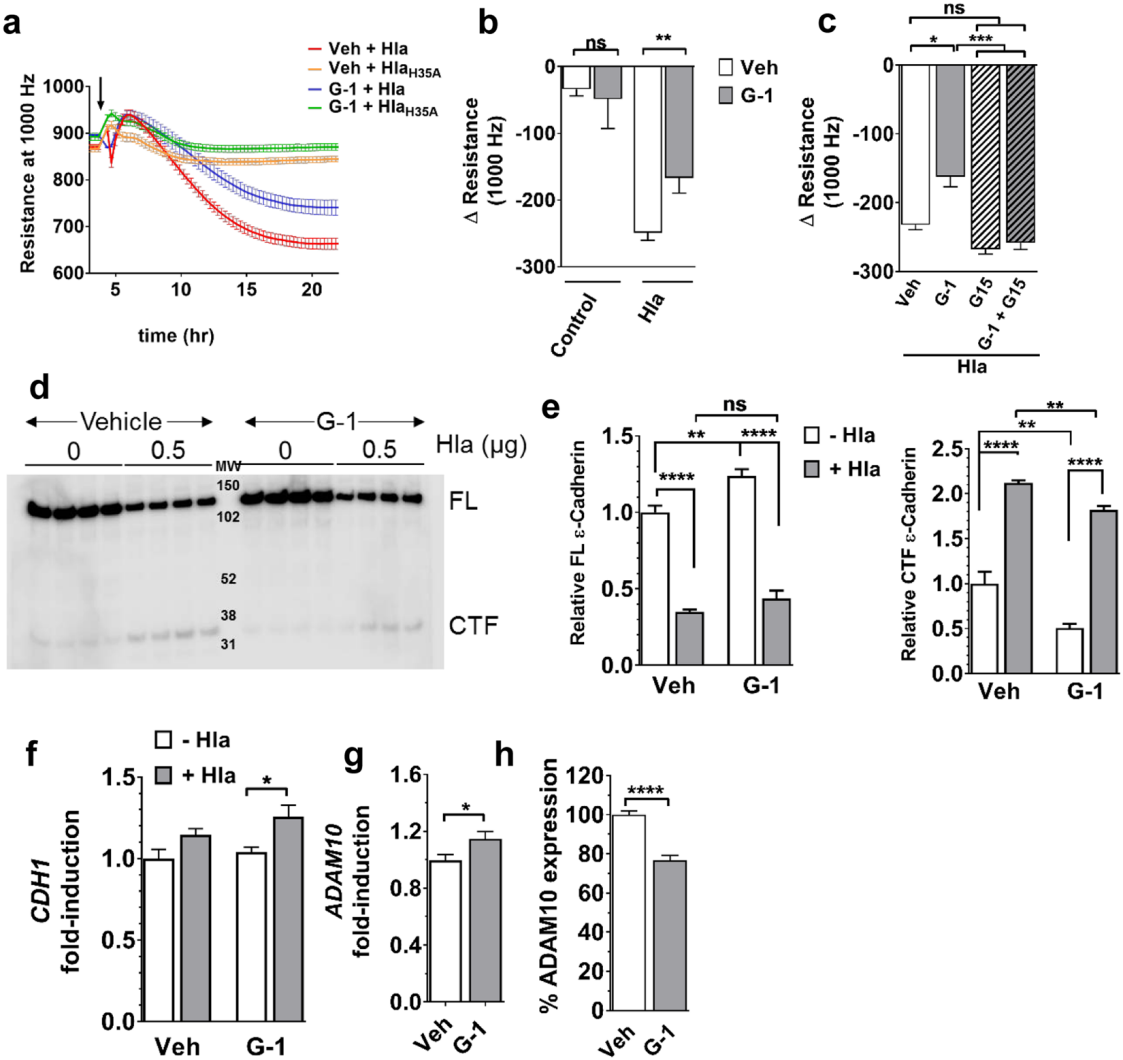
G-1 reduces Hla-mediated keratinocyte permeability barrier disruption. HaCaTs were grown to confluence in the presence of vehicle or 100 nM G-1 prior to challenge (t = 0) with 1 µg/mL Hla or Hla_H35A_ (indicated by arrow in (**a**)). Changes in permeability barrier resistance at 1000 Hz were measured by ECIS. (**a**) Representative ECIS recording of HaCaT monolayer treated as described above. (**b**) Change in HaCaT permeability barrier resistance at 12 h after Hla exposure. Data are mean ± SEM from two (Hla_H35A_) to six (Control and Hla) independent experiments each with 6–8 technical replicates per condition. Mann-Whitney *U* test. (**c**) HaCaT cells were grown in G-1, G15, G-1 + G15 or vehicle control. Shown is the change in permeability barrier resistance at 12 h after Hla exposure. Data are mean ± SEM of a representative experiment of two independent experiments each with a minimum of 8 technical replicates. ANOVA < 0.0001. (**d**) Western blot analysis of full-length (FL) E-cadherin and the cleaved C-terminal fragment (CTF) in vehicle- and G-1-treated (100 nM) HaCaT cell monolayers after eight hours incubation with 0 or 0.5 µg/mL Hla. MW, molecular weight markers. Four replicates for each group are shown. Image Studio Lite was used to invert luminescent image to that shown to uniformly enhance contrast for quantification of CTF. (**e**) Relative quantification of E-cadherin FL (left) and CTF (right) of samples in (**c**) and based on band intensity relative to vehicle-treated cells in the absence of Hla. (**f**) Quantitative PCR analysis of *CDH1* and (**g**) *ADAM10* transcription by vehicle- and G-1-treated (100 nM) HaCaT cells. Expression is shown relative to *GAPDH* and normalized to vehicle-treated control cells. (**h**) Surface expression of ADAM10 on vehicle- and G-1-treated (100 nM) HaCaT cells measured by immunofluorescent staining and flow cytometry. Shown is percent expression relative to vehicle-treated controls. Data are mean ± SEM from two independent experiments each with three technical replicates per condition. Unpaired *t*-test: ns, not significant; *p < 0.05; **p < 0.01; ***p < 0.001; ****p < 0.0001.

Hla is a major contributor to the pathogenesis of *S. aureus* infections involving epithelial cells, including SSTI and pneumonia (PNA)^8,10–13,44–46^. During these infections, Hla disrupts host permeability barriers to facilitate invasive infection^8–14^. Recently, GPER activation was reported to limit disruption of BBB integrity in a rodent model of GCI^35^. Furthermore, GPER is expressed in numerous types of skin cells including melanocytes, dermal fibroblasts and the most prominent skin cell type, keratinocytes^36–39^. Given our *in vivo* data showing that G-1 limits skin damage caused by Hla, and the role of *S. aureus* Hla in disrupting epithelial cell junctions, we hypothesized that activation of GPER with G-1 would limit Hla-mediated epithelial permeability barrier disruption. One measure of permeability barrier integrity is resistance of cell monolayers to passage of an electric current (electrical cell-substrate impedance sensing (ECIS))^47^. As keratinocytes are the major skin cell type, we used the HaCaT human keratinocyte cell line^48^ to determine whether G-1 limits Hla-mediated disruption of epithelial barrier integrity. GPER expression in HaCaT cells was first verified by immunofluorescent staining with and without siRNA knockdown of GPER, after which we demonstrated that G-1 did not affect cell growth or viability during culture (Supplementary Fig. S4). Next, HaCaT cells were grown to confluence in the presence of vehicle or G-1 and changes in transepithelial electrical resistance (TER) were measured by ECIS. After reaching stable resistance, monolayers were exposed to Hla or the inactivate Hla mutant, Hla_H35A_^49,50^. As previously reported, there was no significant reduction in TER between control cells and those challenged with Hla_H35A_^10^ regardless of G-1-treatment (Fig. 4a and data not shown). In contrast, whereas Hla rapidly reduced keratinocyte barrier integrity (decreased TER) in vehicle-treated cells, cells grown in the presence of G-1 were significantly more resistant (p < 0.01) to permeability barrier disruption, with an average 33% increase (p = 0.0079) in TER compared to vehicle control (Fig. 4a,b). The G-1 mediated reduction in permeability barrier disruption was reversed in the presence of the GPER-antagonist, G15^40^ (Fig. 4c), consistent with the requirement for GPER for G-1 efficacy *in vivo* (Fig. 2a–c). Notably, G-1 did not alter HaCaT cell numbers (Supplementary Fig. S4), suggesting that increased barrier integrity is independent of potential G-1-mediated effects on cell growth or viability.

Hla disrupts epithelial barriers by binding its cell surface receptor, ADAM10^10,14,51^, which in turn cleaves E-cadherin. Given that E-cadherin is a component of the adherens junctions responsible for epithelial permeability barrier integrity^10,51^, and that G-1 limited keratinocyte permeability barrier disruption by Hla (Fig. 4a,b) and reduced skin damage (lesion area) in Hla-injected mice (Fig. 3a), we predicted that G-1 would reduce E-cadherin cleavage following Hla challenge. To test this, we used immunoblotting to measure full-length E-cadherin (FL) and the cleaved C-terminal fragment (CTF) from HaCaT cell monolayers grown in the presence of vehicle or G-1 and exposed to Hla (Fig. 4d,e). In the absence of Hla, G-1 significantly increased expression of FL E-cadherin (Fig. 4e, left) while decreasing baseline cleavage (CTF) (Fig. 4e, right). As expected, following an eight-hour culture with Hla, FL E-cadherin was significantly decreased (Fig. 4e, left) and cleavage (CTF) was increased independent of treatment (Fig. 4e, right). However, whereas FL E-cadherin levels were equivalent between vehicle- and G-1-treated cells in the presence of Hla (Fig. 4e, left), G-1-treatment reduced E-cadherin cleavage (CTF) (Fig. 4e, right). Given reduced Hla-mediated E-cadherin cleavage with G-1, we asked whether G-1 altered expression of E-cadherin or the Hla receptor ADAM10. Although transcription of *CDH1*, which encodes E-cadherin, was not altered by G-1 alone, *CDH1* transcription was increased in G-1-treated keratinocytes exposed to Hla (Fig. 4f). Also, whereas G-1 alone increased *ADAM10* transcription approximately 15% (p < 0.05) (Fig. 4g), HaCaT cells treated with G-1 displayed 23% less ADAM10 (p < 0.0001) on the cell surface compared to control-treated cells (Fig. 4h). Together, these findings suggest that G-1 may contribute to transcriptional regulation of *CDH1* when Hla is present, as well as post-transcriptional regulation of ADAM10 expression or trafficking to the keratinocyte cell surface. This in turn may contribute to maintenance of epithelial permeability integrity in the face of Hla-challenge (Fig. 4a,b).

## Discussion

The skin permeability barrier provides protection against transcutaneous water loss, invasion by microbial pathogens and access of environmental toxins to underlying sensitive tissues^4,5^. However, bacteria have had countless generations to evolve powerful tools to disrupt this barrier and cause SSTIs resulting in annual treatment costs of billions of dollars^6^. As the most common cause of SSTI, *S. aureus* secretes Hla to disrupt epithelial barriers and facilitate invasive infection. Specifically, Hla binds ADAM10 on host cells, resulting in cleavage of the cell junction protein E-cadherin and loss of permeability barrier integrity^8–16^. Here we show that G-1, the highly selective ligand of the non-classical estrogen receptor GPER, limits the severity of *S. aureus* SSTI and production of pro-inflammatory cytokines in a murine challenge model. The effects of G-1 are dependent on *S. aureus* expression of Hla, a finding supported by reduced skin pathogenesis (lesion formation) in G-1-treated mice compared to controls following direct Hla challenge. Not surprisingly, G-1 efficacy is dependent upon host expression of GPER, as protection against *S. aureus* SSTI is lost in GPER KO mice. Furthermore, G-1 reduces keratinocyte surface expression of the Hla receptor ADAM10 and limits Hla-mediated disruption of epithelial barrier integrity *in vitro*. Therefore, along with supporting endothelial permeability barrier integrity following ischemic injury^35^, our findings show that G-1 promotes epithelial barrier integrity and host innate defense against a major bacterial toxin. Given that Hla-mediated epithelial injury controls infection outcome^17^, and that G-1 lacks the feminizing effects seen with estrogen treatment^52^, this work demonstrates the potential efficacy of G-1 as an HDT to promote skin innate defense and reduce the burden of *S. aureus* SSTI.

The role of estrogen signaling in immune regulation has historically been studied in terms of the classical estrogen receptors ERα and ERβ^27^. In contrast, although GPER is expressed by a variety of immune cells, including monocytes, macrophages, neutrophils and lymphocytes^31,32,53–60^, the impact of GPER signaling on immune function is in the early stages of investigation. Interestingly, GPER activation can result in both pro- and anti-inflammatory responses. For example, GPER signaling can modulate neutrophil function^31,32^, regulate both pro- and anti-inflammatory cytokine production^30,56^ and promote regulatory T-cell responses^55^. GPER has also been shown to provide protection in a mouse model of multiple sclerosis^30^, to contribute to monocyte-dependent skin inflammation in response to serum from lupus patients, and to reverse peripheral immunosuppression in an ovariectomized mouse model of stroke^33^. GPER is also expressed in skin cells^36–39^, where it contributes to cytoskeletal organization^37,61^ and melanin synthesis^36^. Here we demonstrate the contribution of GPER activation to host innate defense against *S. aureus* skin infection and to increasing epithelial barrier integrity in the face of Hla challenge. These findings not only significantly expand our understanding of GPER signaling in innate immune defense, but also demonstrate a novel role for G-1 in the maintenance of barrier integrity in human keratinocytes.

Hla plays a major role in the pathogenesis of *S. aureus* infection in numerous animal models, particularly models of SSTI and PNA^8,10,11,14–16^. Its importance is further evidenced by ongoing efforts to develop prophylactic and therapeutic strategies targeting Hla expression or function^8,18–25^. For example, recombinant Hla toxoid was recently shown to be a safe and immunogenic candidate vaccine antigen in healthy adults in Phase 1–2 clinical trials (NCT01011335)^25^. In addition, Suvratoxumab (formerly known as MEDI4893), an anti-Hla monoclonal antibody, has successfully completed Phase 1 safety trials (NCT01769417)^62^ and is currently in a Phase 2 safety and efficacy trial (NCT02296320)^63^ to prevent or limit *S. aureus* pneumonia in mechanically ventilated adults. Along with these approaches, the use of G-1, which also protects against Hla-mediated pathogenesis, could provide an additional therapeutic benefit to patients. Interestingly, combination treatment with the Hla neutralizing antibody MEDI4893, and either vancomycin or linezolid, two clinically important antibiotics, improved outcomes in mouse models of *S. aureus* SSTI^18^ and PNA^19,64^. Whether G-1 will have similar adjunctive efficacy with antibiotic therapy against *S. aureus* infection is an important point for future investigation. Further studies are also required to determine the mechanism by which G-1 increases keratinocyte transcription of the gene encoding the Hla receptor ADAM10 while also reducing its surface expression (Fig. 4f,g). Although speculative, this mechanism may involve G-1-mediated effects on post-translational regulation of ADAM10, including suppression of ADAM10 trafficking to the cell surface. In any case, the use of G-1 as an HDT to promote skin innate defense may prove a valuable component of a multifaceted approach to reduce the burden of *S. aureus* infection.

The ability of G-1 to promote epithelial barrier integrity and to limit *S. aureus* infection supports its potential clinical utility in patient populations at increased risk for infection. For example, *S. aureus* colonization is frequently associated with atopic dermatitis and psoriasis, skin diseases that feature epidermal barrier dysfunction^65–68^, and *S. aureus* may actually contribute to skin inflammation in these patients^69^. Recurrent *S. aureus* SSTI is also a hallmark of autosomal dominant hyper IgE syndrome (AD-HIES)^70^ and it has recently been shown that the impaired epithelial response to infection in these patients results from overproduction of the pro-inflammatory cytokine TNFα^71^. TNFα also contributes to the severity of atopic dermatitis, though TNF blockade by anti-TNF biologicals has thus far failed to improve outcomes in these patients^72^. Here we show that whereas G-1 provides greater overall improvement in *S. aureus* skin infection outcomes in the more susceptible male^26^ versus relatively resistant female mouse population, G-1-treatment significantly reduces lesion size as well as TNFα production in *S. aureus* infected mice of both sexes. This suggests that G-1, which reduces but does not completely prevent TNFα production and signaling, may be efficacious in limiting the severity of *S. aureus* skin infection in highly susceptible AD-HIES and atopic dermatitis patients.

Here we used a short-term skin infection model to capture the innate immune response to *S. aureus* infection in advance of the development of any adaptive immunity. Therefore, our findings indicate that G-1 enhances innate immune defense against infection. This suggests the potential of G-1-treatment for limiting infection in groups with increased risk of *S. aureus* infection due to impaired innate immunity such as cancer patients undergoing chemotherapy or surgery^73–76^. Given that what begins as an SSTI can lead to *S. aureus* pneumonia, sepsis or other life threatening infection^77^, it will be essential to experimentally determine the efficacy of G-1 in limiting *S. aureus* skin infection in disease models of patients with increased susceptibility to *S. aureus*. Furthermore, since a single dosing regimen was utilized for the current studies, additional investigations will be needed to determine the optimal dosing strategy for efficacy against *S. aureus* SSTI in immunocompetent mice as well as disease models of susceptible patient populations. Overall, developing an HDT that limits infection in diverse patient populations could positively influence clinical practice and improve human health.

Although our findings suggest the potential utility of G-1 to limit pathogenesis during *S. aureus* skin infection, it could have much broader clinical utility. For example, since many innate defense mechanisms are effective against a variety of bacterial pathogens, therapies aimed at GPER activation may also prove efficacious for treating a variety of infections. In addition, given that a monoclonal antibody targeting Hla has shown synergy with antibiotic therapy in animal models of *S. aureus* infection^18,19,64^, G-1 may likewise have adjunctive efficacy. Furthermore, the ability of G-1 to promote endothelial^35^ and epithelial barrier integrity may prove useful in treating AD, psoriasis, or other diseases involving dysfunctional permeability barriers. Finally, regardless of its therapeutic potential, G-1 may provide a powerful tool for identifying other GPER-dependent host targets to improve epithelial barrier integrity and promote host innate immune defense.

## Methods

### Reagents and cell culture

G-1 was synthesized as previously described^34^. G-1 was dissolved in absolute ethanol to make a 1 mg ml^−1^ stock and stored at −20 °C until use. HaCaT cells were generously provided by Dr. Laurie Hudson (University of New Mexico Health Sciences Center, Albuquerque, NM, USA). Prior to use as described below, HaCaT cells were cultured at 37 °C, 5% CO_2_ in Hyclone^TM^ Dulbecco’s Modified Eagle Medium with low glucose (DMEM low), sodium pyruvate and without phenol red (GE Healthcare, Pittsburgh, PA, USA), plus L-glutamine, 1% Hyclone^TM^ Minimal Essential Media with Non-Essential Amino Acids (MEM NEAA) and 10% FBS (Gibco®, ThermoFisher Scientific, Grand Island, NY, USA).

### Bacterial strains and growth conditions

The MRSA USA300 isolate LAC^41^ and the LAC *hla* deletion mutant (LACΔ*hla*) were provided by Dr. F. DeLeo (Rocky Mountain Laboratories, National Institutes of Health,/National Institute of Allergy and Infectious Diseases, Hamilton, MT) and Dr. J. Bubeck-Wardenburg (Washington University School of Medicine, St. Louis, MO), respectively. For infection, bacteria were cultured in trypticase soy broth (TSB) at 37 °C to early exponential phase as described previously^78^. Stocks were prepared in TSB with 10% glycerol and maintained at −80 °C for no more than two weeks prior to use. The number of CFU per ml of frozen stock was determined by plating ten-fold serial dilutions onto trypticase soy agar containing 5% sheep blood (Becton, Dickinson and Company; Franklin Lakes, NJ, USA). On the day of infection, bacteria were diluted to the indicated concentration in USP-grade saline (B. Braun Medical, Irvine, CA, USA).

### Mouse skin infection model

Male and female C57BL/6 J were purchased from The Jackson Laboratory (Bar Harbor, Maine, USA). Mice were acclimated for a minimum of seven days prior to use. *Gper*^*−/−*^ mice (GPER KO) (Procter & Gamble, Cincinnati, OH, provided by Jan S. Rosenbaum) were backcrossed onto the C57BL/6 background (mice originally purchased from Harlan Laboratories, currently Envigo, Indianapolis, IN, USA) as previously described^52^. *Gper*^*−/−*^and corresponding wild-type (WT) mice (C57BL/6) were bred in-house at the University of New Mexico Animal Resources Facility. The mouse model of SSTI was utilized as previously described^9^. Briefly, four to six days before infection we used Nair™ to depilate the right flank of the mice. The day of use, G-1 stock was diluted in absolute ethanol to 20 µg ml^−1^. Immediately prior to injection, 100 µl of G-1 at 20 µg ml^−1^ in absolute ethanol or 100 µl absolute ethanol control was added to 900 µl diluent (0.9% NaCl with 0.1% bovine serum albumin and 0.1% Tween-20). On days −2 through + 2 relative to infection or Hla challenge, mice were treated by intraperitoneal (IP) injection of 100 µl of vehicle or G-1 (200 ng, ~ 10 µg kg^−1^). On the day of infection (d0), mice (8 to 12 weeks of age) were anesthetized by isoflurane inhalation and infected by subcutaneous (SQ) injection of 50 µl of saline containing 2–3 × 10^7^ CFU of LAC or LACΔ*hla*. Similarly, mice used for Hla challenge studies were injected SQ with 1 µg recombinant Hla^79^. Mice were weighed prior to infection and every 24 hours until sacrifice, and percent weight loss calculated relative to pre-infection weight at 100%. Challenge sites (infection or Hla injection) were photographed daily and lesion areas determined by ImageJ analysis^80^. Lesion area and weight loss over the course of infection was integrated (Prism 8.0.0, GraphPad Software, La Jolla, CA, USA) and presented as area under curve (AUC) in mm^2^. On day 3 post-infection, mice were sacrificed by CO_2_ asphyxiation and a 2.25-cm^2^ section of skin surrounding the infection site was excised for mechanical disruption. As appropriate, skin homogenate was serially diluted and plated on sheep blood agar to determine infection site CFUs. Remaining skin homogenate was clarified by centrifugation and the clarified fraction stored at −80 °C for subsequent analysis.

### Cytokine and MPO analysis

The concentration of the indicated cytokines in clarified skin homogenate was determined using a BioPlex 200 system, BioPlex manager software (Bio-Rad, Hercules, CA, USA) and a custom-designed mouse multiplex assay (EMD Millipore, Billerica, MA, USA) according to manufacturer’s directions. Where cytokine levels were below the limit of detection, one-half of the lowest standard concentration was utilized. Myeloperoxidase (MPO) levels in clarified homogenate were measured using the ELISA Mouse Myeloperoxidase DuoSet kit (R&D Systems, Minneapolis, MN, USA) according to manufacturer’s directions.

### ECIS

TER of HaCaT cell monolayers was measured using an ECIS® Zθ instrument (Applied Biophysics, Troy, NY, USA). Cells were maintained at 37 °C, 5% CO_2._ ECIS 96W10idf disposable electrode arrays (Applied Biophysics) were coated with 25 µl of 0.01% poly-L-lysine (Millipore Sigma, Burlington, MA, USA) and incubated at least 15 min. Poly-L-lysine was then removed and replaced with media A (250 µl DMEM low without phenol red, with sodium pyruvate, L-glutamine, 1% MEM-NEAA and 10% charcoal-stripped FBS (JR Scientific, Woodland, CA, USA)) and TER was measured every 10 min at 1000 Hz. After resistance stabilization, media A was removed and replaced with 300 µl of HaCaT cells (6.3 × 10^4^) in prewarmed media A containing 100 nM G-1, 1 µM G15, 100 nM G-1 plus 1 µM G15 or vehicle control ( + G-1/G15/Veh). Cells were allowed to stabilize for six days with media replaced on day 3. On day 5, media was changed to media B (DMEM high glucose, without phenol red, with sodium pyruvate, L-glutamine and 1% MEM-NEAA) + G-1/G15/Veh. On day 6, media was replaced with media B + G-1/G15/Veh containing either 1 µg Hla, Hla_H35A_ or PBS (phosphate buffered saline) control, and TER measures continued for 24 hours.

### Quantitative PCR

Infection site tissue (2.25-cm^2^) was collected in RNALater (Qiagen, Valencia, CA), RNA isolated using Qiazol and purified using RNeasy Kits (Qiagen, Germantown, MD, USA) according to manufacturer’s directions. cDNA was generated from RNA using a PTC-200 Peltier Thermocycler (Bio-Rad, Hercules, CA, USA) with High-capacity cDNA RT kits with RNAse inhibitor and random hexamer primers (Applied Biosystems, Foster City, CA, USA). Quantitative PCR (qPCR) was performed on a ViiA 7 Real-Time PCR system (Applied Biosystems) using Taqman® Gene Expression master mix (Applied Biosystems). Gene expression was quantified using QuantStudio software (Applied Biosystems) relative to *GAPDH* using Prime Time Predesigned qPCR assays for *CDH1* and *ADAM10* (Integrated DNA Technologies, Coralville, IA, USA).

### Immunofluorescent staining and flow cytometry

HaCaTs were seeded in 24-well plates (4 × 10^5^ cells in one ml in media A with G-1/Veh) and replaced as needed until confluent. Media was changed to media B + G-1/Veh 24 hours prior to flow analysis. HaCaTs were trypsinized (0.25% Trypsin-EDTA, Gibco) and cells pooled from either G-1- or Veh-treated wells. Pooled cells were subsequently washed and exchanged into flow buffer (PBS, 2% FBS, and 0.1% NaN_3_) then diluted to equal cell concentrations (~3 × 10^6^ cells ml^−1^). Fc inhibitor antibody (14-9161-71, ThermoFisher) was added at 20 µl ml^−1^ and cells incubated at room temperature (RT) for 20 min. Cells were then stained on ice in the dark for 30 min with either isotype control (12-4714-81, ThermoFisher) or anti-ADAM-10 (352703, BioLegend, San Diego, CA) antibody at 10 µg ml^−1^. Cells were washed three times with flow buffer and 20,000 events were recorded on an Accuri C6 flow cytometer (BD Biosciences, San Diego, CA) with isotype corrected mean fluorescence normalized to Veh-treated controls.

### Western blot analysis of E-cadherin cleavage

Throughout this assay, all media included either 100 nM G-1 or vehicle control (G-1/Veh) as described above and cells were maintained at 37 °C, 5% CO_2_. HaCaTs were seeded in 24-well plates (4 × 10^5^ cells in one ml of media A + G-1/Veh) and media was replaced every 48 hours until cells were confluent. Cells were then grown for 24 hours in media B + G-1/Veh. After 24 hours, cells were treated for eight hours with 0.5 µg ml^−1^ Hla (or PBS) in media B + G-1/Veh. To collect both cleaved E-cadherin (CTF), which is released intracellularly, and full-length E-cadherin (FL), which is cell-associated, an equal volume of 2X RIPA lysis buffer^81^ (Triton X-100 was substituted for Nonidet P-40) was added to each well and the plate incubated on ice for five min. Equal volumes of lysate were resolved on 4–12% Bis-Tris Plus gels in MES buffer (ThermoFisher) and transferred to 0.45 µm nitrocellulose membranes (Bio-Rad, Hercules, CA). Membranes were blocked with 5% nonfat milk in TBST (20 mM Tris, pH 7.6, 150 mM NaCl, and 0.1% Tween 20) for 90 min at RT. E-cadherin was detected using mouse anti-human E-cadherin antibodies (FL-sc-8426, Santa Cruz Biotechnology, Dallas, TX; CTF - 610181, BD Biosciences, San Diego, CA) at 1:1000 followed by goat anti-mouse poly-HRP antibody (32230, ThermoFisher), both in 1% milk in TBST. Membranes were developed using SuperSignal West Femto Substrate (ThermoFisher), and imaged on a Protein Simple FluorChem R system (ProteinSimple, Santa Jose, CA). Band intensity was quantitated using Image Studio Lite (v5.2, LI-COR, Lincoln, NE) and normalized to vehicle-treated, no Hla lysates.

### Statistical analysis

GraphPad Prism version 7.03 (GraphPad Software, San Diego California) was used for all statistical evaluations. Statistical analyses of two groups was performed using an Unpaired Students *t*-test or Mann Whitney *U* test for non-parametrics as appropriate based on data normality. For comparisons between more than two groups, one-way ANOVA was used with parameters based on D’Agostino & Pearson omnibus or Shapiro-Wilk normality tests, and with Tukey’s or Bonferroni’s (ANOVA) or Dunn’s (Kruskal-Wallis test, non-parametrics) post-hoc multiple comparison analyses as indicated. Results were considered statistically significant at p < 0.05.

### Ethics approval

All animal studies were conducted in adherence with the recommendations in the *Guide for the Care and Use of Laboratory Animals*^82^ and the Animal Welfare Act, and were approved by the Institutional Animal Care and Use Committee (IACUC) of the University of New Mexico Health Sciences Center (Animal Welfare Assurance number D16-00228).

## Supplementary information

Supplementary Information

## Data Availability

The datasets generated during the current study are available from the corresponding author on reasonable request.
