# Supplementary material for: GPER activation protects against epithelial barrier disruption by Staphylococcusaureus α-toxin
Source: Sci Rep. 2019 Feb 4;9:1343. doi: 10.1038/s41598-018-37951-3 (PMC6362070; doi:10.1038/s41598-018-37951-3)
Supplement: Supplementary file 1 — Supplementary Information [file 41598_2018_37951_MOESM1_ESM.pdf]

GPER activation protects against epithelial barrier disruption by *Staphylococcus aureus* α-toxin

Kathleen D. Triplett[1+], Srijana Pokhrel[1,+], Moriah J. Castleman[1], Seth M. Daly[1], Bradley O. Elmore[1], Jason A. Joyner[1], Geetanjali Sharma[2], Guy Hebert[1], Matthew J. Campen[1], Helen J. Hathaway[3], Eric R. Prossnitz[2] and Pamela R. Hall[1,*]

[1]University of New Mexico College of Pharmacy, Department of Pharmaceutical Sciences, Albuquerque, NM 87131, USA

[2]University of New Mexico School of Medicine, Department of Internal Medicine, Albuquerque, NM 87131, USA

[3]University of New Mexico School of Medicine, Department of Cell Biology & Physiology, Albuquerque, NM 87131, USA

SUPPLEMENTAL METHODS

***S. aureus* growth assay.** An overnight culture of LAC was diluted to 5×10^5 CFU ml^{-1} and treated with 1 µM or 100 nM G-1 or vehicle control. Treated bacteria were aliquoted to a 96-well plate sealed with a breathable membrane (USA Scientific, Ocala, FL). Plates were incubated at 37ºC with shaking, and the OD_{600} was measured every 15 min for 16 hours using a Synergy HT plate reader (BioTek, Winooski, VT).

Hla Western blot and relative quantification. Overnight cultures of LAC and LACΔ*hla* were diluted 1:100, treated with 100 nM G-1 or vehicle and incubated at 37°C with shaking for 18 hours. The bacteria were pelleted and the supernatant filtered through a 0.2 µm polyethersulfone filter (VWR, Radnor, PA). Protein concentrations were determined by bicinchoninic acid assay (BCA) (ThermoFisher) and equal protein volumes were resolved on a 4-12% Bis-Tris Plus gel in MES buffer (ThermoFisher) before transfer to a 0.45 µm nitrocellulose membrane (Bio-Rad, Hercules, CA). Membranes were blocked with 5% nonfat milk in TBS (20 mM Tris, pH 7.6, and 150 mM NaCl) for 2 hours at RT. Hla was detected with mouse anti-HLA antibody (6C12, IBT Bioservices, Rockville, MD) at 1:1000 and goat anti-mouse poly-HRP antibody (32230, ThermoFisher) at 1:10,000, both in 1% milk in TBS. TBST (TBS plus 0.1% Tween 20) was used for membrane washes between incubations. Membranes were developed in SuperSignal West Femto Substrate (ThermoFisher), and imaged using a Protein Simple FluorChem R (ProteinSimple, Santa Jose, CA). Band intensity was quantitated using ImageStudio Lite (v5.2, LI-COR, Lincoln, NE) and normalized to vehicle-treated, no Hla lysates.

Rabbit red blood cell lysis assays. To assess G-1 effects on Hla production and activity, glycerol stocks of LAC were diluted to 2 x 10^7 CFU ml^{-1} and either left untreated or treated with 1 µM and 100 nM G-1 or vehicle then incubated at 37ºC with shaking for 5 hours. Bacteria were pelleted by centrifugation and the supernatant filtered through a 0.2 µm polyethersulfone filter (VWR). To determine the effects of G-1 on Hla function, G-1 or vehicle was added to previously untreated cultures just prior to supernatant harvest as described above. Supernatants were diluted 2-fold in PBS and incubated statically with an equal volume of PBS-washed, 4% rabbit red blood cells (31081, Colorado Serum Company, Denver, CO) at 37ºC. Lysis was measured as a decrease in turbidity at OD_{650} using a SpectraMax 340 plate reader (Molecular Devices, San Jose, CA).

HaCaT cell count and viability. HaCaTs (300 µl with 6.3 x 10^4 total cells) were seeded into a 96-well plate in media A + G-1/Veh and media replaced every 48 hours until the cells were confluent. Cells were then grown for 24 hours in media B +G-1/Veh before being trypsinized (0.25% Trypsin-EDTA, Gibco) and diluted 1:1 with 0.4% trypan blue (Gibco). Cells were counted using a TC20 automated cell counter (Bio-Rad). Viable cells were those that excluded trypan blue.

Immunofluorescence and siRNA for GPER expression. Human keratinocyte HaCaT cells were seeded on coverslips for 2-3 days, washed and fixed in 2% paraformaldehyde. For some experiments, cells on coverslips were transfected with human GPER siRNA (Dharmacon L-005563-00) or non-targeting control siRNA (Dharmacon D-001810-10-05) using Lipofectamine 3000 (Thermo Fisher Scientific) as per manufacturer's instructions 48 hours before fixing. For staining GPER, cells were permeabilized and blocked with 3% BSA in PBST (PBS containing 0.1% Triton X-100) for 1 h and then incubated with rabbit anti-human GPER antibody (SAB

2700363, Sigma at 1:250), using 3% normal goat serum (NGS) in PBS as blocker for 3 h. Cells were washed with PBS and incubated with goat anti-rabbit IgG conjugated to Alexa Fluor 568 in PBS containing 3% NGS for 1 h. Subsequently, cells were washed, mounted in Vectashield, and imaged by confocal microscopy using a Zeiss LSM800 Airyscan confocal microscope (Zeiss, Oberkochen, Germany).

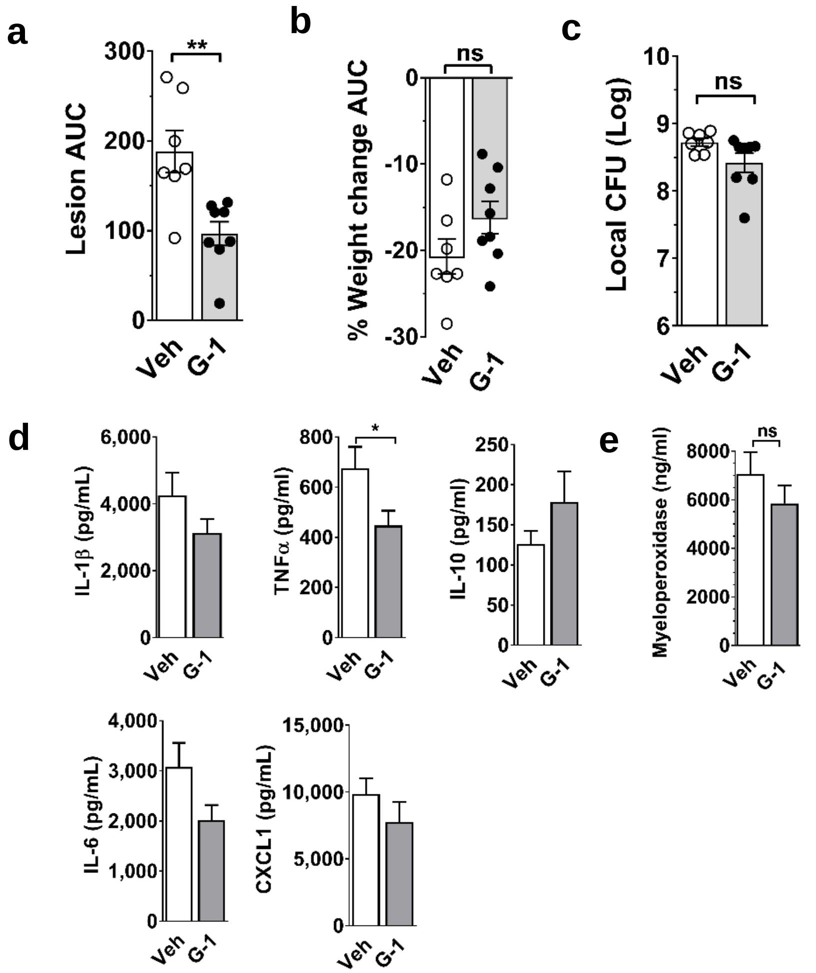

Supplementary Figure S1. G-1 promotes protection against SA SSTI in female mice. WT (C57BL/6J) female mice were treated IP with vehicle (Veh) or 200 ng G-1 on days -2, -1, 0, +1 and +2 relative to SQ infection with 3 x 10^7 CFU of USA300 MRSA isolate LAC. Mice were weighed and lesion size measured daily before mice were sacrificed on day 3 post-infection. (**a**) AUC for lesion size (mm^2) and (**b**) percent weight change over the 3-day infection. (**c**) Day 3 post-infection bacterial burden. (**d**) IL-1β, TNFα, IL-6, CXCL1 and IL-10 levels in clarified injection site homogenate collected on day 3 from the site of infection. (**e**) Day 3 post-infection myeloperoxidase levels in clarified infection site homogenate. n=7-8 mice per group from two independent experiments. Data are mean ± SEM Unpaired *t*-test: ns, not significant; *, $p<0.05$; **, $p<0.01$.

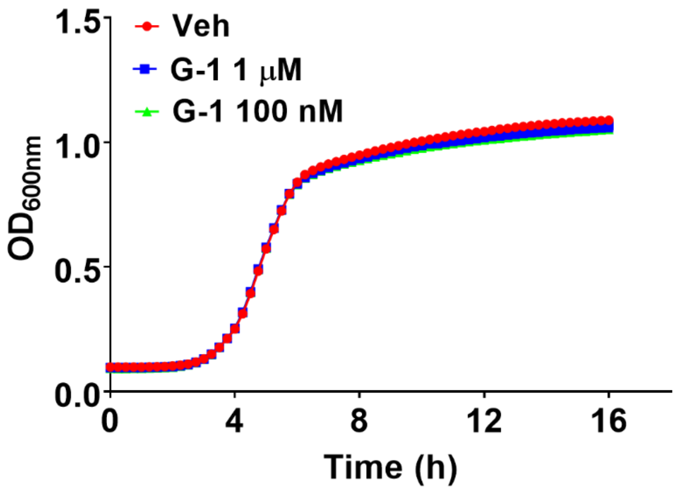

Supplementary Figure S2. G-1 does not inhibit *S. aureus* growth. Growth of LAC in the presence of 100 nM and 1 μM G-1 or vehicle control as measured by OD_{600}. Data are mean ±SEM.

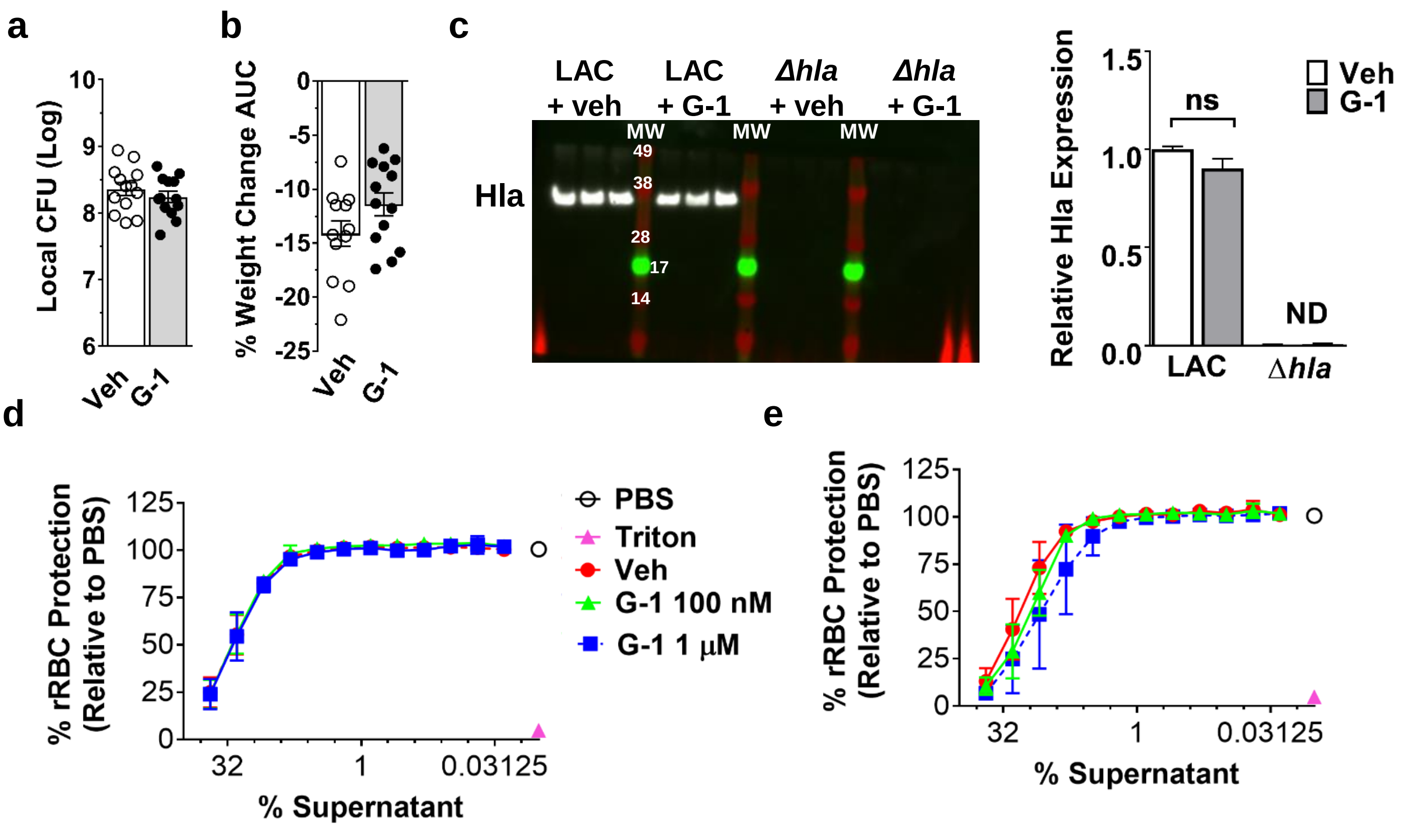

Supplementary Figure S3. G-1 does not alter SSTI outcomes in the absence of Hla and does not inhibit Hla production or activity. **(a-b)** WT (C57BL/6J) male mice were treated IP with vehicle or 200 ng G-1 on days -2, -1, 0, +1 and +2 relative to SQ infection with 3 x 10^7 CFUs LACΔ*hla*. (**a**) Day 3 post-infection bacterial burden and (**b**) percent weight change (AUC) over the 3 day infection. Data are mean ± SEM, n=13 mice per group from two independent experiments. **(c)** Western blot (left) and quantification (right) of Hla in sterile supernatant from LAC and LACΔ*hla* grown for 18 h in the presence of vehicle control or 100 nM G-1. Three samples per group are shown (left). ND, not detected. **(d-e)** Analysis of Hla-mediated rabbit red blood cell (rRBC) lysis using sterile LAC supernatant collected after 5 h growth in the presence of the indicated concentrations of G-1 or vehicle control **(d)** or sterile 5 h LAC supernatant with the indicated concentrations of G-1 or vehicle control added at the time of the assay (**e**). Percent protection against rRBC lysis relative to PBS at 100%. Triton control shows total lysis. Data are mean ± SD. Unpaired *t*-test; ns, not significant.

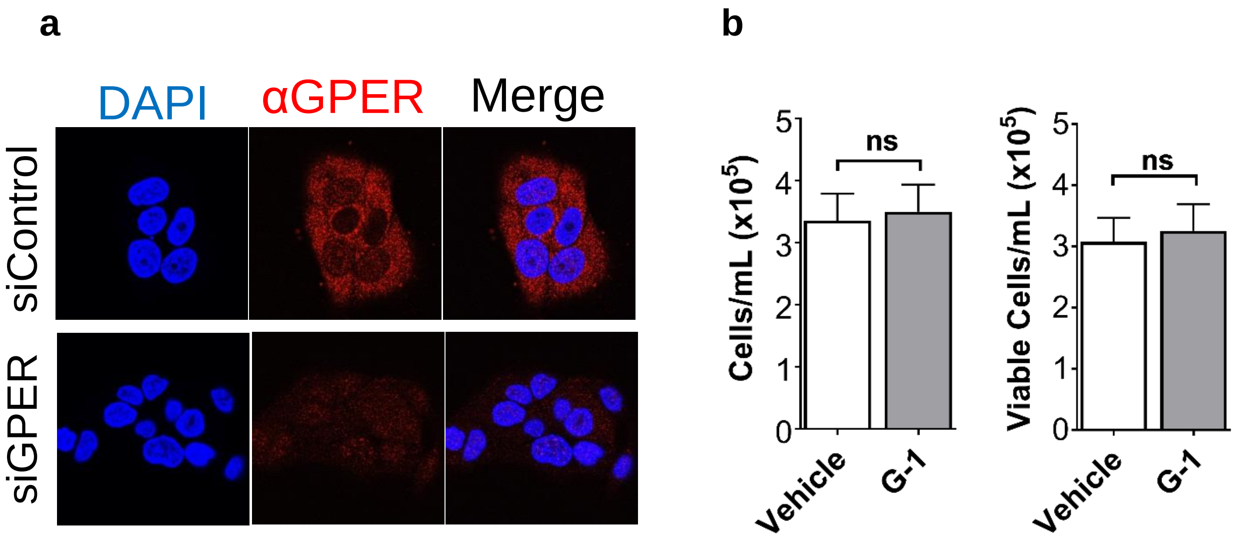

Supplementary Figure S4. GPER is expressed in HaCaT cells and G-1 does not alter HaCaT cell growth or viability. (a) Immunofluorescence microscopy showing GPER staining in siControl-treated HaCaT cells, but not in siGPER-treated cells. **(b)** Cell count and viability of HaCaT cells grown to confluence in the presence of 100 nM G-1 or vehicle. Data are mean ± SEM. Unpaired *t*-test. ns, not significant.
